# All-cause hospitalizations for inflammatory bowel diseases: Can the reason for admission provide information on inpatient resource use? A study from a large veteran affairs hospital

**DOI:** 10.1186/s40779-016-0098-x

**Published:** 2016-09-06

**Authors:** Ashish Malhotra, K.C. Mandip, Aasma Shaukat, Thomas Rector

**Affiliations:** 1 Division of Gastroenterology, Department of Medicine, Minneapolis VA Medical Center, University of Minnesota, Minneapolis, MN 55147 USA; 2 Center of Innovation, Minneapolis VA Medical center, Minneapolis, MN USA; 3 Department of Medicine, University of Minnesota, Minneapolis, MN USA

**Keywords:** Inflammatory bowel diseases, Veteran affairs, Readmission rate

## Abstract

**Background:**

Inflammatory bowel diseases (IBDs) are group of chronic inflammatory illnesses with a remitting and relapsing course that may result in appreciable morbidity and high medical costs secondary to repeated hospitalizations. The study’s objectives were to identify the reasons for hospitalization among patients with inflammatory bowel diseases, and compare inpatient courses and readmission rates for IBD-related admissions *versus* non-IBD-related admissions.

**Methods:**

A retrospective chart review was performed on all patients with IBD admitted to the Minneapolis VA Medical Center between September 2010 and September 2012.

**Results:**

A total of 111 patients with IBD were admitted during the 2-year study period. IBD flares/complications accounted for 36.9 % of the index admissions. Atherothrombotic events comprised the second most common cause of admissions (14.4 %) in IBD patients. Patients with an index admission directly related to IBD were significantly younger and had developed IBD more recently. Unsurprisingly, the IBD admission group had significantly more gastrointestinal endoscopies and abdominal surgeries, and was more likely to be started on medication for IBD during the index stay. The median length of stay (LOS) for the index hospitalization for an IBD flare or complication was 4 (2–8) days compared with 2 (1–4) days for the other patients (*P =* 0.001). A smaller percentage of the group admitted for an IBD flare/complication had a shorter ICU stay compared with the other patients (9.8 % *vs.* 15.7 %, respectively); however, their ICU LOSs tended to be longer (4.5 *vs. *2.0 days, respectively, *P =* 0.17). Compared to the other admission types, an insignificantly greater percentage of the group whose index admission was related to an IBD flare or complication had at least one readmission within 6 months of discharge (29 % *versus* 21 %; *P =* 0.35). The rate of admission was approximately 80 % greater in the group whose index admission was related to an IBD flare or complication compared to the other types of admission (rate ratio 1.8, 95 % confidence interval 0.96 to 3.4), although this difference did not reach statistical significance (*P =* 0.07).

**Conclusion:**

Identifying the reasons for the patients' index admission, IBD flares *versus* all other causes, may provide valuable information concerning admission care and the subsequent admission history.

## Background

Inflammatory bowel diseases (IBDs) are a group of chronic inflammatory illnesses with a remitting and relapsing course that may result in appreciable morbidity and high medical costs secondary to repeated hospitalizations. The clinical management of IBD patients has improved due to increased understanding of the disease process and new therapies. However, disease morbidity remains high, with an estimated mean annual direct health care cost per IBD patient in the US of roughly $8265 and $5066 for Crohn’s disease (CD) and ulcerative colitis (UC), respectively. Approximately 31 and 38 % of these costs, respectively, are directly attributed to acute hospitalization [1]. Within the Department of Veterans Affairs (VA), there is an increasing burden of disease related to IBD. A recent study using VA administrative data found that the prevalence of Crohn’s disease and ulcerative colitis had increased 2- to 3-fold among veterans between 1998 and 2009 [2]. The VA population differs from the general US population due to combat exposure, male predominance, lower socio-economic status, and higher psychiatric comorbidities [3], all of which can potentially affect IBD progression and treatment. There is a paucity of data on inpatient resource utilization by veterans with IBD. We sought to identify the reasons for hospitalization among patients with IBD and to compare the inpatient course and readmission rates for IBD-related admissions versus non-IBD-related admissions. To this end, we conducted a clinical review of all patients admitted to the tertiary care VA hospital in Minneapolis, MN, USA, during the fiscal years 2010 and 2011 to assess the reasons for hospitalization, the inpatient course and readmissions.

## Methods

A retrospective chart review was conducted of all patients with an IBD diagnosis admitted to the Minneapolis VA Medical Center between September 2010 and September 2012. Ethics approval was granted for the chart review by the Institutional Review Board at the Minneapolis VAMC.

Patients were identified by the International Classification of Diseases version 9 (ICD-9) diagnostic codes representing Crohn’s disease (555.×) and ulcerative colitis (556.×). Hospitalization data were identified for 132 unique patients. Subjects with missing data on the index hospitalization or patients determined not to have IBD on chart review (*n =* 21) were excluded.

The patients’ demographics and disease histories were obtained from the hospitalization and clinic notes in the electronic medical records. The principle diagnosis for each hospitalization was ascertained by a physician based on the review of hospitalization records. The criteria for defining an IBD flare/complication were as follows: any hospitalization requiring steroids or other widely accepted treatments for IBD, such as tumor necrosis factor (TNF) inhibitors, and surgical procedures related to the intestinal tract. In-hospital resource use was determined by LOS, intensive care unit use (ICU) and recurrent hospitalizations within 6 months.

### Statistical analyses

Continuous variables were described by the mean and standard deviation of the distribution unless they were highly skewed, in which case the results were described using medians and interquartile ranges (IQRs). Depending on the level of measurement and the data distributions, these groups were compared using a *t*-test, rank sum test or Chi-square test. Six-month hospital readmission rates per 100 patient-months were estimated and compared using Poisson regression.

## Results

A total of 111 IBD patients were admitted during the 2-year study period. IBD flares/complications accounted for 41 (36.9 %) of the index admissions. Other reasons for admission included atherothrombotic events (14.4 %), non-GI infections (9.0 %), orthopedic surgery (6.3 %), oncological reasons (5.4 %), arrhythmia (4.5 %), renal reasons (acute kidney injury on chronic kidney disease 2/2 FTT, hemodialysis complications) (5.4 %), pulmonary reasons (COPD, tracheobronchitis) (3.6 %), psychiatric reasons (2.7 %), and miscellaneous causes (other surgeries, non-stroke neurological reasons, elective procedure requiring admission, giardiasis) (11.8 %). Table 1 compares the characteristics of the patients whose index admissions were for an IBD flare or complication versus patients whose admissions were not directly related to IBD. Patients with an index admission directly related to IBD were significantly younger and had developed IBD more recently. Approximately half of each group had a psychiatric co-morbidity. Unsurprisingly, the IBD admission group had significantly more gastrointestinal (GI) endoscopies and abdominal surgeries and was more likely to be started on medications for IBD during the index stay. The median LOS (interquartile range) for the index hospitalizations for an IBD flare or complication was 4 (2–8) days, whereas the LOS was 2 (1–4) days for the other patients (*P =* 0.001). A smaller percentage of the group admitted for an IBD flare or complication had an ICU stay [4/41 (9.8 %) vs. 11/70 (15.7 %)]; however, their ICU LOSs tended to be longer [4.5d (2–6.5d) vs. 2d (1–2d), respectively; *P =* 0.17]. There were no in-hospital deaths.

**Table 1:** Baseline characteristics of patients by reason for index hospital admission

Item	IBD flare or complication (*n =* 41)	Other type of admission (*n =* 70)
Mean age (year)	54.5 ± 16.9**	64.7 ± 12.4
Male (n)	40	70
Year of index admission (n)		
2010	7*	17
2011	12*	33
2012	22*	20
Type of IBD (n)		
Ulcerative colitis	20	34
Crohn’s disease	21	36
IBD duration [n (%)]		
New	2 (4.9)*	0 (0)
< 1 year	3 (7.3)*	1 (1.4)
> 1 year	35 (85.4)*	61 (87.1)
Unknown	1 (2.4)*	8 (11.4)
Psychiatric co-morbidity [n (%)]	23 (56.1)	31 (44.3)
Inpatient procedures [n (%)]		
GI endoscopy	9 (22.0)*	5 (7.1)
Abdominal surgery	9 (22.0)*	4 (5.7)
Non-abdominal surgery	0**	15 (21.4)
Medications initiated in the hospital [n (%)]		
Antibiotics	10 (24.4)**	3 (4.3)
Corticosteroids	14 (34.1)**	2 (2.9)
Thiopurines	2 (4.9)**	0
Biologics	2 (4.9)**	0
Discharge Medications		
New medications added	37 (90.2)**	14 (20.0)
Changed dose	3 (7.3)**	1 (1.4)
New pain medications added	15 (36.6)**	6 (8.6)
Existing medications discontinued	7 (17.1)**	4 (5.7)

*P < 0.05, **P < 0.001

All-cause readmissions within 6 months after discharge from the index admission are summarized in Table 2. Compared to other types of admissions, an insignificantly greater percentage of the group whose index admission was related to an IBD flare or complication had at least one readmission within 6 months of discharge (29.2 % *vs* 21.4 %; *P =* 0.35). The rate of admission was approximately 1.8 times greater in the group whose index admission was related to an IBD flare or complication compared to the other types of admissions (rate ratio 1.8, 95%CI 0.96–3.40), although this difference did not reach statistical significance (*P =* 0.07).

**Table 2:** Readmissions within 6 months by reason for index hospital admission

Item	IBD flare or complication (*n =* 41)	Other type of admission (*n =* 70)
Number of readmissions in 6 months [n (%)]		
0	29 (70.7)	55 (78.6)
1	7 (17.1)	12 (17.1)
2	3 (7.3)	2 (2.9)
3	1 (2.4)	1 (1.4)
4	1 (2.4)	0
Median time to first readmission in months (IQR)	0.6 (0.3–3.1)	1.3 (0.3–4.0)
Number of patients readmitted [*n* (%)]	12 (29.3)	15 (21.4)
Total number of readmissions (*n*)	20	19
Total person-months ^a^	242	414
Incidence rate of readmission (95 % CI) per 100 person-months ^b^	8.3 (5.2–3.1)	4.6 (2.8–0.3)

^a^ Calculated by multiplying *n* to 5.92 (as data was collected in number of days)

^b^ Rate ratio is calculated as the ratio between the two incidence rates of readmission per 100 person-months

*IQR* interquartile range, *CI* confidence interval

## Discussion

Interventions designed to reduce the frequency of hospitalizations and the attendant medical costs of IBD patients require understanding the factors driving the admission of these patients. To obtain these data, we analyzed the reasons for hospitalization and the utilization of inpatient healthcare resources by US veterans with IBD. In this analysis, it became apparent that identifying the reason for the patient’s index admission (IBD flare versus all other causes) provided valuable information concerning admission care and the subsequent admission history.

Several key findings deserve emphasis. First, out of the 111 IBD patients admitted to the hospital over a 2-year period, only approximately 1/3 of the admissions were for problems directly related to IBD; the other 2/3 of the patients were admitted for a variety of non-IBD medical problems, most commonly atherothrombotic conditions. Because gastroenterologists tend to see patients with active IBD, problems with IBD flares tend to monopolize the attention of the physicians. These admission data emphasize the frequency of non-IBD problems and the need for care directed to the entire gamut of medical conditions rather than a concentration simply on gastrointestinal problems. Of particular interest was the frequency of atherothrombotic events in our patients with quiescent IBD. Previous studies have found an increased incidence of venous and arterial thrombotic events in IBD patients [4], which may reflect the pro-thrombotic state that has been postulated to be caused by low-grade systemic inflammation [5].

Secondly, there was a strikingly increased prevalence of opiate usage by patients admitted with a flare (17 %) vs. only 4 % of patients admitted for non-IBD problems. The 17 % prevalence seems high relative to Targownik et al., [6] who reported a maximum of 11 % of IBD subjects with an active opioid prescription in the first month following diagnosis, with this usage falling as the disease process came under control. Several factors have been shown to be associated with outpatient opioid use in IBD populations, including psychiatric comorbidities, such as depression and anxiety, a history of substance abuse, female gender, and clinical disease activity when measured by symptoms [7, 8]. Almost half of our cohort had psychiatric comorbidities, which might account for the higher prevalence of opioid use in this population. Prior studies have also shown that opioid use may be associated with more severe disease [9, 10] and may be independently related to poor outcomes [9], although the cause or effect relationship remains to be determined. Finally, there are reports showing an overall increased prevalence of opioid use in veteran populations, although opiate use in veterans with IBD has not been previously studied [11].

Third, we found that patients admitted for IBD flares were more likely to have a longer LOS (both in the ICU and overall). The average LOS reported by Kappelman et al. [1] was 6.7 days for CD and 6.9 days for UC, although this study did not identify the reasons for hospitalization. The median LOS in our study was 4.0 days for an IBD flare and 2.0 days for other admissions, which was comparable to a recent Swiss study showing a mean number of hospitalization days of 2.0 (±8.8) and 1.5 (±6.1) for CD and UC, respectively. While it is tempting to speculate that the shorter hospitalizations reflect recent improvements in the medical management of IBD, confirmation of this speculation requires additional study. It is well established that hospitalization for IBD exacerbations can be resource-intensive, primarily due to the number of surgical procedures performed in this group [1]. We also found that patients admitted for IBD-related reasons were significantly more likely to undergo endoscopic and abdominal surgical procedures.

Fourth, patients with IBD-related admissions were more likely to have significant changes in their medication profiles upon discharge from the hospital compared to patients with non-IBD-related admissions. This issue raises concern over the possibility that a lack of appreciation of such changes among patients or poor communication of such changes to the outpatient providers could result in disease mismanagement and potential readmissions or urgent care visits. A randomized clinical trial demonstrated that readmissions to hospitals were often related to deficiencies in coordination and communication within the health care system and with patients. Thus, we speculated that good transitional care interventions to systematically address communication and care-coordination and to promote self-management in patients admitted for IBD flares/complications could be of significant value.

The limitations of our study merit attention. We relied on available clinical and administrative data to assess and account for differences in illness severity at the patient or hospital level. Thus, residual confounding cannot be ruled out. Additionally, we were unable to ascertain the necessity of hospitalization or the criteria used to decide to readmit. It is possible that we underestimated the rate of readmission if the veteran was re-hospitalized at a different institution. Finally, the generalizability of our study may be limited by the clinical setting in which the observations were made. Some potential variability in patient populations served by other VA facilities may limit the generalizability of our findings.

## Conclusion

This study highlights the importance of identifying the reason for hospitalization among IBD patients. Patients admitted for IBD-related reasons are more resource intensive in terms of length of stay and use of surgical procedures. Additionally, there was a tendency towards a higher readmission rate in patients admitted for IBD-related problems opposed to the other patients, although the difference did not reach statistical significance. This issue may require confirmation with a larger sample size. Finally, whether interventions focusing on care-coordination and communication at the time of transition of care (i.e., discharge from the hospital) reduce the readmission rate in IBD patients should be addressed in a future study.

## Abbreviations

CD: Crohn’s disease
FTT: Failure to thrive
GI: Gastrointestinal
IBD: Inflammatory bowel disease
ICD: International classification of diseases
ICU: Intensive care unit
IQR: Interquartile range
LOS: Length of stay
TNF: Tumor necrosis factor
UC: Ulcerative colitis
VA: Veterans affairs
VAMC: VA medical center

## References

[1] Kappelman M, Rifas-Shiman S, Porter C, Ollendorf D, Sandler R, Galanko J, Finkelstein J (2008). Direct health care costs of Crohn’s disease and ulcerative colitis in US Children and adults. Gastroenterology.

[2] Hou JK, Kramer JR, Richardson P, Mei M, El-Serag HB (2013). The incidence and prevalence of inflammatory bowel disease among U.S. veterans: A national cohort study. Inflamm Bowel Dis.

[3] Kazis LE, Miller DR, Clark J, Skinner K, Lee A, Rogers W (1998). Health-related quality of life in patients served by the department of veterans affairs. Arch Intern Med.

[4] Miehsler W, Reinisch W, Valic E, Osterode W, Tillinger W, Feichtenschlager T (2004). Is inflammatory bowel disease an independent and disease specific risk factor for thromboembolism?. Gut.

[5] Giannotta M, Tapete G, Emmi G, Silvestri E, Milla M (2015). Thrombosis in inflammatory bowel diseases: what’s the link?. Thromb J.

[6] Targownik LE, Nugent Z, Singh H, Bugden S, Bernstein CN (2014). The prevalence and predictors of opioid use in inflammatory bowel disease: A population-based analysis. Am J Gastroenterol.

[7] Hanson KA, Loftus EV, Harmsen WS (2009). Clinical features and outcome of patients with inflammatory bowel disease who use narcotics: a case-control study. Inflamm Bowel Dis.

[8] Edwards JT, Radford-Smith GL, Florin THJ (2001). Chronic narcotic use in inflammatory bowel disease patients: Prevalence and clinical characteristics. J Gastroenterol Hepatol.

[9] Cheung M, Khan S, Akerman M, Hung CK, Vennard K, Hristis N (2015). Clinical markers of Crohn’s disease severity and their association with opiate use. J Clin Med Res.

[10] Cross RK, Wilson KT, Binion DG (2005). Narcotic use in patients with Crohn’s disease. Am J Gastroenterol.

[11] Baser O, Xie L, Mardekian J, Schaaf D, Wang L, Joshi AV (2014). Prevalence of diagnosed opioid abuse and its economic burden in the veterans health administration. Pain Pract.

